# Selection of *Streptomyces* against soil borne fungal pathogens by a standardized dual culture assay and evaluation of their effects on seed germination and plant growth

**DOI:** 10.1186/s12866-016-0886-1

**Published:** 2016-11-09

**Authors:** Andrea Kunova, Maria Bonaldi, Marco Saracchi, Cristina Pizzatti, Xiaoyulong Chen, Paolo Cortesi

**Affiliations:** Department of Food, Environmental and Nutritional Sciences (DeFENS), Università degli Studi di Milano, via Giovanni Celoria, 2, 20133 Milan, Italy

**Keywords:** Dual culture assay, Antagonism, Antibiosis, Biological control, Seed colonization, Plant growth promotion

## Abstract

**Background:**

In the search for new natural resources for crop protection, streptomycetes are gaining interest in agriculture as plant growth promoting bacteria and/or biological control agents. Because of their peculiar life cycle, in which the production of secondary metabolites is synchronized with the development of aerial hyphae and sporulation, the commonly used methods to screen for bacterial antagonists need to be adapted.

**Results:**

The dual culture assay was standardized in terms of inoculation timing of *Streptomyces* antagonist and pathogen, and growth rate of different fungal pathogens. In case of fast-growing fungi, inoculation of the antagonist 2 or 3 days prior to the pathogen resulted in significantly stronger inhibition of mycelium growth. One hundred and thirty *Streptomyces* strains were evaluated against six destructive soil borne pathogens. The activity of strains varied from broad-spectrum to highly specific inhibition of individual pathogens. All strains inhibited at least one tested pathogen. Three strains, which combined the largest broad-spectrum with the highest inhibition activity, were selected for further characterization with four vegetable species. All of them were able to colonize seed surface of all tested vegetable crops. They mostly improved radicle and hypocotyl growth in vitro, although no statistically significant enhancement of biomass weight was observed in vivo. Occasionally, transient negative effects on germination and plant growth were observed.

**Conclusions:**

The adapted dual culture assay allowed us to compare the inhibition of individual *Streptomyces* strains against six fungal soil borne pathogens. The best selected strains were able to colonize the four vegetable crops and have a potential to be developed into biocontrol products. Although they occasionally negatively influenced plant growth, these effects did not persist during the further development. Additional in vivo studies are needed to confirm their potential as biological control or plant growth promoting agents.

**Electronic supplementary material:**

The online version of this article (doi:10.1186/s12866-016-0886-1) contains supplementary material, which is available to authorized users.

## Background

Modern agricultural systems aim to ensure sustainable crop production and thus they drive the search for new natural resources to find environmentally friendly solutions for crop protection and yield increase. As a part of this research, streptomycetes, a group of Gram-positive, mycelium-forming bacteria are gaining interest in agriculture as plant growth promoting bacteria (PGPB) and/or biological control agents (BCAs). The genus *Streptomyces* comprises more than 500 species ubiquitous in nature, which can be free-living in soil or symbionts of eukaryotic organisms, ranging from fungi to plants, insects and marine animals (reviewed in [1]). Few *Streptomyces* species are pathogens of tuberous and taproot crops causing economically important diseases [2], and very little is known about the effects of other species on plant growth and health. Some streptomycetes are able to colonize plant rhizosphere and live as endophytes in the roots of numerous crops [3–6]. Occasional reports demonstrated that *Streptomyces* species were able to suppress plant pathogens, and some of them promoted plant growth [7, 8]. They protected plant roots by inhibiting fungal pathogens through the production of lytic enzymes and antifungal compounds [9–13]. Local and systemic activation of plant defense systems through induced or acquired resistance by *Streptomyces* was described, in which they primed the defense pathways by inducing low-level expression of systemic acquired resistance and jasmonate/ethylene pathways [14–16]. Furthermore, plant growth promotion was observed through the auxin or siderophore production. Positive effects of *Streptomyces* application on seed germination and on root and shoot growth were reported [17–23]. These observations, the ability of *Streptomyces* to produce a huge variety of antimicrobial secondary products [24] and their presence in soil make them promising candidates for the selection and development as plant protection products and biofertilizers.

The selection of biological control agents usually starts with an in vitro screening of a collection of strains against selected pathogens by a dual culture assay, in which the candidate BCA is co-cultivated with the pathogen on agar medium and its antagonistic activity is quantified in terms of inhibition of pathogen’s mycelium growth [25]. The production of many secondary metabolites in *Streptomyces* is induced upon the development of aerial hyphae and the beginning of sporulation [26–28]. However, this developmental peculiarity was not always considered in previous studies, where the inoculation timing of the *Streptomyces* strains varied from inoculating *Streptomyces* 7 days before the pathogen to the co-inoculation at the same day [29–32]. Moreover, the growth rate of different pathogens was also not taken into consideration. Therefore, there is a strong need for a standardized methodology that would consider the particular life cycle of *Streptomyces* antagonists and growth rate of different fungal pathogens. This will help to reduce the risk of producing biased results when screening a large pool of streptomycetes against a variety of fungal pathogens. Similarly to the biocontrol studies, the selection of PGP strains involves in vitro screening for compounds such as phytohormones and siderophores, followed by in vivo evaluation of their activity [32–34]. Diverse *Streptomyces* spp. were shown to produce PGP related metabolites and enhance plant growth through phosphate solubilization, iron chelation, and auxin production [22, 35–37]. Indeed, positive effects of streptomycete application on seed germination, root growth, as well as hypocotyl development were observed [17–21, 23].

Biological control and PGP are two different but linked components associated with beneficial plant-microbe interactions [38], and can share the same molecular mechanisms. For instance, some fungal pathogens require iron for their pathogenicity [39]. On the other hand, siderophores produced by beneficial rhizobacteria are responsible for scavenging ferric iron from the environment and may directly inhibit the pathogen growth by iron competition [40]. At the same time, they make the iron available for plant growth and may serve as inducers of plant systemic resistance [34, 41, 42]. Sometimes, negative effects of streptomycetes on plant growth were also observed, e.g., some *Streptomyces* strains adversely influenced the growth of monocotyledonous and dicotyledonous plants [43, 44]. Therefore, evaluating effects of streptomycetes on plant growth in absence of pathogen is an important step in developing *Streptomyces*-based biocontrol products.

The objectives of this work were, 1) to standardize the dual culture assay considering the inoculation timing of *Streptomyces* and the pathogen growth rate, 2) to evaluate the standardized method by screening the antagonism of 130 root endophytic streptomycetes against six common and destructive soil borne fungal pathogens of vegetable crops, and 3) to evaluate the effects of three most active streptomycete strains on seed germination and growth of four vegetable crops.

## Results

### Mycelium growth rate of selected soil borne fungal pathogens

**Fig. 1:**
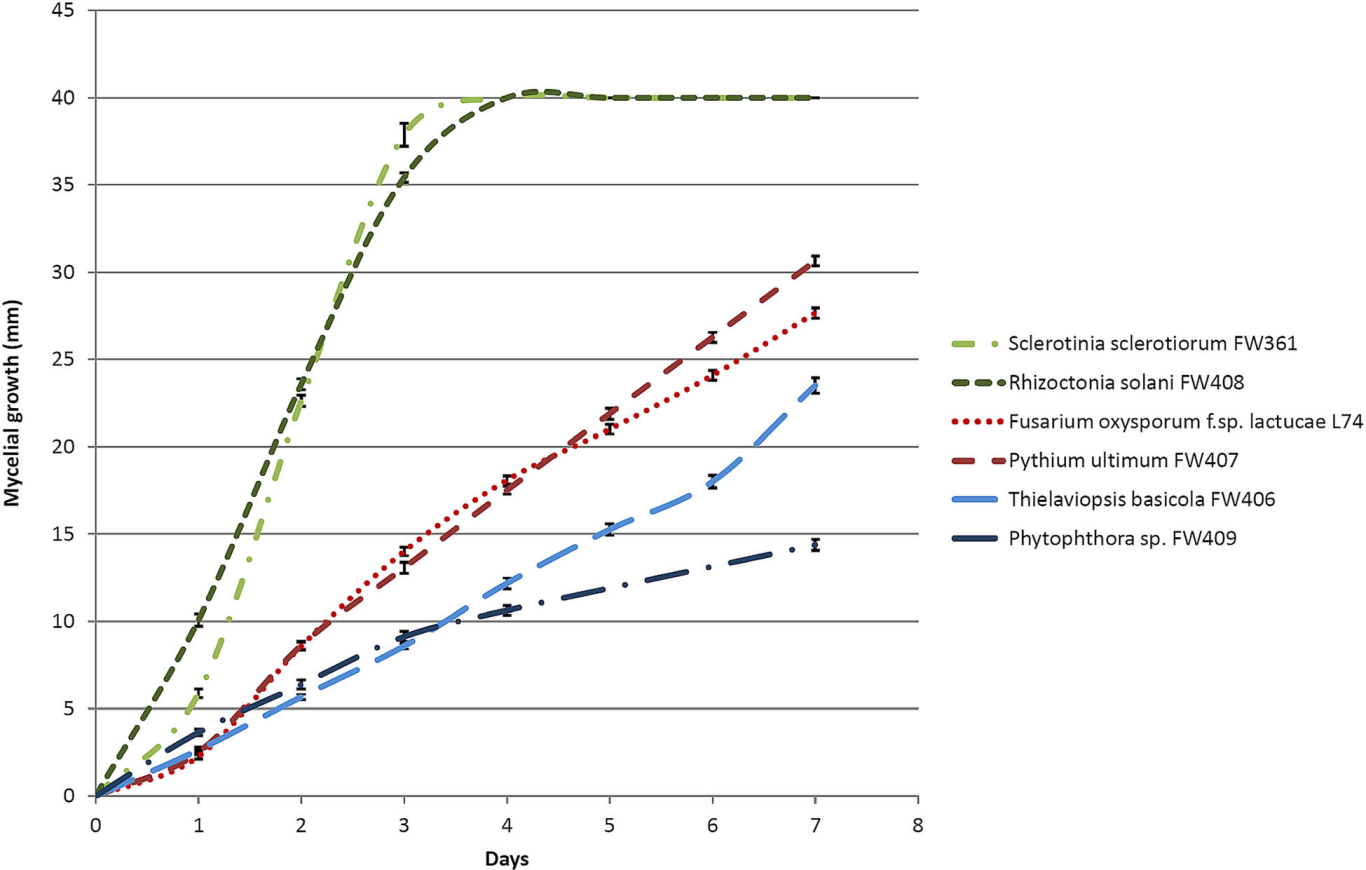
Mycelial growth of six tested soil borne fungal pathogens. The pathogens were divided into three groups based on their growth rate: fast-growing (*green*), medium-growing (*red*) and slow-growing (*blue*). The error bars represent the standard error

The growth curves of the six soil borne fungal pathogens were determined on CZY medium: *Sclerotinia sclerotiorum* FW361, *Rhizoctonia solani* FW408, *Fusarium oxysporum* f.sp. *lactucae* L74, *Pythium ultimum* FW407, *Phytophthora* sp. FW409 and *Thielaviopsis basicola* FW406 (Fig. 1). Based on their growth rate, they were divided into three groups: 1. fast-growing, 2. medium-growing, and 3. slow-growing. The group 1 comprised *S. sclerotiorum* FW361 and *R. solani* FW408; their colonies reached the edges of the 90 mm Petri plates 4 days after the inoculation. *F. oxysporum* f.sp. *lactucae* L74 and *P. ultimum* FW407 grew slightly slower: 4 days after inoculation, their mean radial growth was 17.5 and 18.1 mm, respectively and were classified as group 2. The group 3 included *Phytophthora* sp. FW409 and *T. basicola* FW406, which grew very slowly; 4 days after the inoculation their mean radial growth was 10.6 and 12.2 mm, respectively.

### Optimization of the dual culture assay

The dual culture assay was optimized to be able to compare the inhibition activity of 130 *Streptomyces* strains against six soil borne fungal pathogens belonging to the three groups based on their mycelium growth rate. We standardized the inoculation timing of pathogen and *Streptomyces* and the distance between the microorganisms. First, the inoculation timing of the two microorganisms was investigated. Two *Streptomyces* reference strains (*S. rochei* CMJ57I and *S. anulatus* CX14W) were inoculated 3, 2 or 1 day before or at the same day as *S. sclerotiorum* FW361 (group 1) or *F. oxysporum* f.sp. *lactucae* L74 (group 2) and the inhibition of pathogen mycelium growth was assessed 3 and 7 days after pathogen inoculation, respectively. In case of *S. sclerotiorum* FW361, when *Streptomyces* was inoculated 2 or 3 days before the pathogen, it caused significantly stronger inhibition of mycelium growth, while for *F. oxysporum* f.sp. *lactucae* L74 the inhibition of mycelium growth was not significantly different regardless the timing of streptomycete inoculation (Table 1). Next, to be able to compare the effect of the antagonists on fast- and slow-growing fungi, the distance between the two microorganisms was set as the mean of the 4-day-mycelial growth for the pathogens of the groups 2 and 3; 20 mm and 10 mm respectively; and the antagonist and the pathogen were inoculated the same day. The pathogens of group 1 were inoculated 2 days after the antagonist to allow the production of bio-active compounds by streptomycetes and were placed in the distance corresponding to 2-day-mycelial growth (25 mm). The inhibition of mycelial growth was assessed 3 days after pathogen inoculation for fungi in the group 1, and 6 and 7 days after inoculation for fungi in groups 2 and 3, respectively.

**Table 1:** Inhibition of pathogen mycelium growth based on timing of *Streptomyces* inoculation

		Day of *Streptomyces* inoculation^a^	Mycelium growth inhibition (%)	
			*Sclerotinia sclerotiorum* FW361	*Fusarium oxysporum* f. sp. *lactucae* L74
*Streptomyces* strain	CMJ57I	0	58.43^b^ b^c^	56.06 a
		−1	67.12 b	56.06 a
		−2	77.35 a	58.82 a
		−3	80.99 a	56.06 a
	CX14W	0	63.50 b	59.41 a
		−1	71.61 ab	55.71 a
		−2	74.65 a	55.88 a
		−3	74.57 a	60.29 a

^a^ Day 0 refers to the co-inoculation of the pathogen and *Streptomyces*, Days −1, −2 and −3 correspond to inoculation of *Streptomyces* 1, 2 or 3 days before the pathogen

^b^ mean value of mycelium growth inhibition

^c^ Tukey *post-hoc* test; mean values in a column with the same letters are not significantly different (*P* = 0.05)

### Selection of streptomycetes based on their antagonistic activity

After the optimization of the dual culture assay, the antagonistic activity of 130 *Streptomyces* strains was evaluated. They showed a wide range of inhibition activity against individual pathogens (Additional file 1: Table S1). The activity against *S. sclerotiorum* FW431 ranged from 0 to 87 %, with *Streptomyces* sp. ALP07R being the most active strain. The activity against *R. solani* FW408 was lower and varied from 0 to 72.4 %, with *Streptomyces* sp. SLF27R being the best strain. *F. oxysporum* f.sp. *lactucae* L74 was the least inhibited pathogen: *Streptomyces* sp. SLF27R, the most active strain, inhibited its growth by 47.9 %; and 15 out of 130 strains did not show any activity against this pathogen. The activity against *P. ultimum* FW407 varied from 0 to 63.64 % and St*reptomyces* sp. CX08W was the best strain. Similarly, *Streptomyces* sp. MR01W was the most active against *T. basicola* FW406 and *Phytophthora* sp. FW409, with inhibition activity of 85.7 and 67.4 %, respectively. The mean rank values were calculated for each *Streptomyces* strain as described in Materials and Methods and the first three strains were chosen for further studies: *S. cyaneus* ZEA17I, *S. anulatus* CMJ58I and *S. albidoflavus* VT111I (Table 2). *S. cyaneus* ZEA17I was the most active strain: it strongly inhibited especially *S. sclerotiorum* FW431 (79.5 %) and *R. solani* FW408 (67.5 %). *S. anulatus* CMJ58I was very active against *T. basicola* FW406 (76.2 %), *P. ultimum* FW407 (57.4 %) and *Phytophthora* sp. FW409 (74.4 %). Also *S. albidoflavus* VT111I resulted very active against *P. ultimum* FW407 (55.9 %), but not against *Phytophthora* sp. FW409 (26.0 %)

**Table 2:** Mycelial growth inhibition of six fungal soil borne pathogens by *Streptomyces cyaneus* ZEA17I, *S.anulatus* CMJ58I and *S. albidoflavus* VT111I and their respective mean rank positions following dual culture assay

*Streptomyces* sp.	Mean rank position	Mycelium growth inhibition (%)					
		*Sclerotinia sclerotiorum* FW361	*Rhizoctonia solani* FW408	*Fusarium oxysporum* f.sp. *lattucae* L74	*Thielaviopsis basicola* FW406	*Pythium ultimum* FW407	*Phytophtora* sp. FW409
ZEA17I	12.0	79.49	67.54	34.06	51.79	50.74	62.00
CMJ58I	21.8	46.79	41.75	28.99	76.19	57.35	74.42
VT111I	27.3	64.10	53.95	28.47	52.38	55.88	26.00

### Scanning electron microscope (SEM) studies of streptomycete seed colonization

**Fig. 2:**
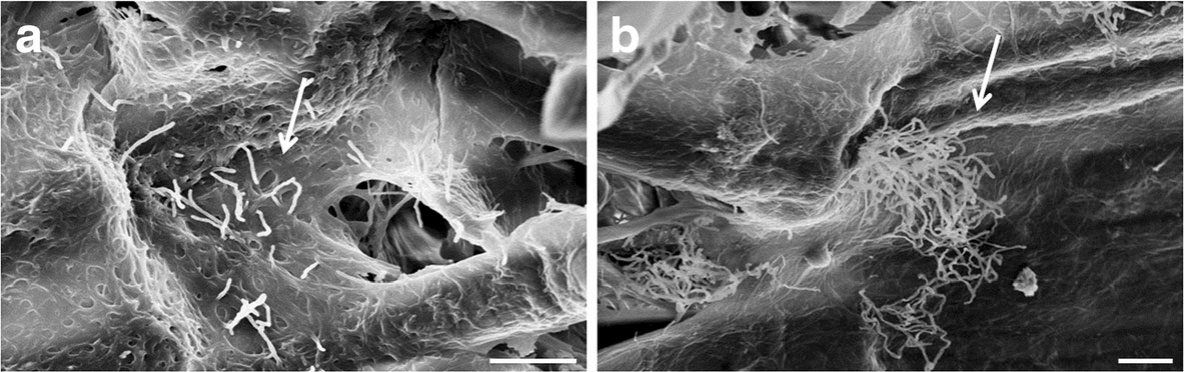
Scanning electron microscopy micrographs of tomato seed surface 24 h (**a**) and 72 h (**b**) after inoculation with *Streptomyces albidoflavus* VT111I. Arrows indicate the hyphae and the mycelium of *S. albidoflavus* VT111I. Bar equals to 10 μm

Prior to evaluating the PGP activity of the three selected *Streptomyces* strains, the colonization of seed surface of lettuce, lamb lettuce, rocket and tomato was examined by SEM. The observations confirmed that seed surfaces of all tested vegetable crops, previously inoculated with streptomycetes, were successfully colonized. The hyphae of *Streptomyces* strains were observed as early as 24 h post inoculation (hpi), and 72 hpi seed surface was abundantly colonized (Fig. 2). Untreated seeds did not show any colonization (data not shown).

### Seed germination

The germination of seeds of cultivated rocket, lamb lettuce, lettuce and tomato inoculated with *S. cyaneus* ZEA17I, *S. anulatus* CMJ58I and *S. albidoflavus* VT111I was assessed both in vitro and in vivo (Table 3). No differences in germination were observed in vitro, while some differences were observed in vivo. The selected strains did not show any negative effects on germination of cultivated rocket and lettuce. However, the germination of lamb lettuce was significantly inhibited after the bacterization with *S. albidoflavus* VT111I (33.0 %) compared to untreated control (64.0 %). Similarly, inhibition of tomato germination was observed after the treatment with *S. cyaneus* ZEA17I (88.3 %) compared to untreated control (96.0 %).

**Table 3:** In vitro (a) and in vivo (b) seed germination of cultivated rocket, lamb lettuce, lettuce and tomato untreated and treated with *Streptomyces anulatus* CMJ58I, *S. albidoflavus* VT111I and *S. cyaneus* ZEA17I

	Days of incubation	Seed treatment			
		Untreated control	*S. anulatus* CMJ58I	*S. albidoflavus VT111I*	*S. cyaneus* ZEA17I
a) In vitro germination (%)					
Cultivated rocket	6	84.3 ± 4.63^a^ ns^b^	80.0 ± 2.00 ns	85.0 ± 1.73 ns	74.3 ± 7.22 ns
Lamb lettuce	10	88.7 ± 2.96 ns	90.7 ± 1.76 ns	87.0 ± 3.61 ns	85.0 ± 3.06 ns
Lettuce	7	79.7 ± 2.85 ns	78.3 ± 2.40 ns	81.7 ± 1.86 ns	84.3 ± 1.86 ns
Tomato	8	95.0 ± 1.53 ns	96.0 ± 1.15 ns	96.3 ± 2.19 ns	96.7 ± 1.33 ns
b) In vivo germination (%)					
Cultivated rocket	10	65.7 ± 8.09 ns	80.0 ± 1.15 ns	78.3 ± 2.19 ns	58.7 ± 5.36 ns
Lamb lettuce	14	64.0 ± 0.58 a	58.7 ± 4.06 a	33.0 ± 6.43 b	44.0 ± 4.04 ab
Lettuce	10	76.0 ± 1.00 ns	71.0 ± 1.15 ns	71.0 ± 3.06 ns	70.7 ± 4.37 ns
Tomato	10	96.0 ± 1.00 a	97.7 ± 0.33 a	92.0 ± 2.52 ab	88.3 ± 1.33 b

^a^ mean value followed by standard error

^b^ Tukey *post-hoc* test; mean values in a row with the same letters are not significantly different (*P* = 0.05); *ns* not significant

### Seedling root and hypocotyl length

The root and hypocotyl lengths of seedlings grown in vitro varied depending on the combination vegetable crop - streptomycete strain (Table 4). All three strains significantly promoted the growth of radicles and hypocotyls of cultivated rocket and tomato and the radicles of lamb lettuce; e.g. *S. anulatus* CMJ58I augmented radicle growth of cultivated rocket ca. three-times (46.83 mm compared to 15.52 mm in untreated control). Moreover, *S. anulatus* CMJ58I promoted also the growth of lamb lettuce hypocotyls by ca. 30 % and *S. cyaneus* ZEA17I significantly enhanced hypocotyl growth of lettuce (3.51 mm compared to 2.97 mm in untreated control). However, in lamb lettuce, *S. albidoflavus* VT111I and *S. cyaneus* ZEA17I negatively affected the radicle growth by 35 and 46 % respectively; and *S. cyaneus* ZEA17I also significantly reduced hypocotyl length by 18 %.

**Table 4:** Effect of *Streptomyces* sp. on root and hypocotyl length of seedlings of cultivated rocket, lamb lettuce, lettuce and tomato in vitro

	Length (mm)	Seed treatment			
		Untreated control	*S. anulatus* CMJ58I	*S. albidoflavus* VT111I	*S. cyaneus* ZEA17I
Cultivated rocket	Radicle	15.52 ± 1.00^a^ c^b^	46.83 ± 2.11 a	31.11 ± 1.79 b	29.77 ± 2.05 b
	Hypocotyl	9.34 ± 0.31 b	11.53 ± 0.36 a	11.11 ± 0.34 a	11.75 ± 0.40 a
	N	253	239	255	222
Lamb lettuce	Radicle	16.79 ± 0.51 b	26.35 ± 0.74 a	11.00 ± 0.33 c	9.08 ± 0.42 c
	Hypocotyl	11.28 ± 0.37 b	14.55 ± 0.34 a	10.41 ± 0.30 bc	9.24 ± 0.37 c
	N	266	272	261	255
Lettuce	Radicle	16.33 ± 0.68 d	39.22 ± 1.95 b	28.31 ± 1.50 c	58.59 ± 2.10 a
	Hypocotyl	2.97 ± 0.06 b	2.77 ± 0.05 b	2.95 ± 0.06 b	3.51 ± 0.06 a
	N	239	235	245	253
Tomato	Radicle	54.17 ± 1.09 b	59.33 ± 1.45 a	63.96 ± 1.55 a	63.41 ± 1.00 a
	Hypocotyl	11.52 ± 0.26 c	13.41 ± 0.33 b	14.69 ± 0.32 a	14.79 ± 0.29 a
	N	285	288	289	290

^a^ The mean value followed by standard error

^b^ Tukey *post-hoc* test; mean values in a row with the same letters are not significantly different (*P* = 0.05); *ns* not significant

### Biomass dry weight in vivo

The strain-specific effects on plant biomass of four vegetable crops were observed also in vivo in controlled conditions (Table 5). No significant differences were observed among biomass dry weight of any vegetable crop, when the three strains were compared to control. Interestingly, *S. cyaneus* ZEA17I increased the dry weight of 2-week-old tomatoes compared to *S. anulatus* CMJ58I and *S. albidoflavus* VT111I. Instead, in 3-week-old lettuce, *S. anulatus* CMJ58I showed negative effect on biomass compared to *S. cyaneus* ZEA17I and *S. albidoflavus* VT111I.

**Table 5:** Effect of *Streptomyces* sp. on plant dry weight of cultivated rocket, lamb lettuce, lettuce and tomato after 2 (a) and 3 (b) weeks of growth in vivo

Dry weight (mg)	Seed treatment			
	Untreated control	*S. anulatus* CMJ58I	*S. albidoflavus* VT111I	*S. cyaneus* ZEA17I
a) 2 weeks				
Cultivated rocket	5.13 ± 0.31^a^ ns^b^	5.42 ± 0.06 ns	4.73 ± 0.24 ns	5.58 ± 0.14 ns
Lamb lettuce	3.91 ± 0.23 ns	4.6 ± 0.22 ns	3.93 ± 0.26 ns	4.65 ± 0.26 ns
Lettuce	3.32 ± .25 ns	3.16 ± 0.12 ns	3.14 ± 0.18 ns	3.66 ± 0.16 ns
Tomato	5.58 ± 0.08 ab	5.35 ± 0.21 b	4.95 ± 0.48 b	6.64 ± 0.26 a
b) 3 weeks				
Cultivated rocket	101.88 ± 22.71 ns	65.95 ± 6.79 ns	88.28 ± 4.24 ns	70.80 ± 4.34 ns
Lamb lettuce	33.69 ± 1.39 ns	31.08 ± 1.49 ns	35.44 ± 2.27 ns	28.15 ± 2.62 ns
Lettuce	76.02 ± 4.48 ab	57.55 ± 4.21 b	79.37 ± 5.77 a	77.62 ± 5.91 a
Tomato	43.62 ± 6.35 ns	48.38 ± 5.15 ns	56.47 ± 4.31 ns	45.58 ± 6.93 ns

^a^ The mean value followed by standard error. At 2 weeks (a) the mean value was calculated as the dry weight of 50 plantlets in three replicates, except for lamb lettuce, where 30 plantlets were collected. At 3 weeks (b) the mean value was calculated as the average dry weight of 20 individually grown plantlets

^b^ Tukey *post-hoc* test; mean values in a row with the same letters are not significantly different (*P* = 0.05); *ns* not significant

### Production of secondary metabolites

All three tested strains produced indole-3-acetic acid. The highest amount was produced by *Streptomyces anulatus* CMJ58I (4.25 μg/mL, s.e. 0.063), whereas *S. cyaneus* ZEA17I and *S. albidoflavus* VT111I produced 3.71 μg/mL (s.e. 0.17) and 3.26 μg/mL (s.e. 0.16), respectively. None of the strains produced siderophores.

## Discussion

Over the years, diverse bacteria - such as *Pseudomonas* spp. and *Bacillus* spp. - have been tested as BCAs, mostly studying the antagonism as the mechanism of action to suppress pathogen growth through the production of antifungal compounds [40, 45]. Recently, another group - streptomycetes - are of interest in agriculture as PGPB and BCAs, as they are commonly found in soil and are able to colonize rhizosphere and root tissues [3, 33, 46]. They surely have the potential to act as antagonists against a variety of soil borne pathogens, as they are renowned for the production of bioactive secondary metabolites. However, until now they have been exploited mostly in pharmaceutical industry for the production of antibiotics, antitumorals or immunosuppressives [47, 48].

The most commonly used method for high-throughput screening of antagonistic microorganisms against fungal pathogens in vitro is the dual culture assay [25]. However, streptomycetes are peculiar bacteria characterized by mycelial growth and particular life cycle, where the production of bioactive secondary metabolites is coordinated with the switch to the formation of aerial hyphae and spores [26, 28, 49]. Therefore, the aim of this work was to improve and adapt the dual culture assay for screening the antagonistic activity of streptomycetes. We particularly considered the coordination of antibiotic production with the *Streptomyces* developmental program, similarly to Schrey et al. [28], who used the onset of *Streptomyces* sporulation as the time for fungus inoculation in co-culture. Moreover, we put the emphasis on the variable mycelium growth rate of the studied pathogens, so that we would be able to select a pool of strains showing a broad spectrum antagonistic activity against multiple fungal pathogens.

In the first step, we grouped the pathogens based on their mycelium growth rate into three groups and we tested the importance of pathogen growth rate and inoculation timing of both microorganisms in the dual culture assay. In the case of fast-growing fungi such as *S. sclerotiorum* we observed a significantly higher inhibition when *Streptomyces* was inoculated 2–3 days before the pathogen, which was not confirmed for slower growing fungi. This observation is in line with Schrey et al. [28] and confirms that *Streptomyces* start to produce the bioactive secondary metabolites later during their life cycle, and that, especially in the case of fast-growing pathogens, they need to be applied prior the pathogen inoculation, as we observed previously in vivo, where *Streptomyces* application 7 days before lettuce sowing significantly reduced *Sclerotinia* drop [9, 50].

The modified dual culture assay was applied to a collection of 130 streptomycetes and allowed us to compare the inhibition activity of individual *Streptomyces* strains against six fungal soil borne pathogens. We selected three potential wide-spectrum bio-active strains, *S. anulatus* CMJ58I, *S. albidoflavus* VT111I and *S. cyaneus* ZEA17I, showing strong in vitro activity against all six pathogens. The results of the dual culture assay emphasized the antagonist-pathogen specific activity of individual *Streptomyces* strains. These results indicate that diverse streptomycetes use different antagonistic mechanisms and produce different bioactive molecules involved in the antagonism against different pathogens.

In order to develop strains showing biocontrol potential into commercial products, they need to be able to interact with the host to exert their activity in vivo [18]. Therefore, we studied the three selected streptomycetes for their ability to colonize various vegetable crops. The observations of seed surface by scanning electron microscopy revealed that all the three strains were able to grow on all tested hosts as early as 24 hai, as previously observed by Coombs and Franco [51] on wheat seeds inoculated with a *Streptomyces* sp. strain EN27. Moreover, *S. cyaneus* ZEA17I was observed on the surface and inside of 2-week-old lettuce roots by confocal and SEM microscopy [50]. Therefore, *S. anulatus* CMJ58I, *S. albidoflavus* VT111I and *S. cyaneus* ZEA17I are valuable candidates as beneficial bacteria for plant growth promotion and disease prevention.

It is also important that the potential BCAs do not affect negatively the germination and growth of the host plant. Among the variety of secondary metabolites, *Streptomyces* often produce IAA, which can improve the plant growth by stimulating cell elongation and root growth [18]. All the three tested strains produced IAA at high concentrations compared to previous studies [52], and the amount of IAA might be further increased in certain conditions [53]. Some authors observed improvement of plant growth by IAA-producing streptomycetes, for instance *Streptomyces* sp. CMUH009 promoted maize seed germination by 20 % compared to control seeds treated with sterile water [54]. Similarly, the three strains, *S. anulatus* CMJ58I, *S. albidoflavus* VT111I and *S. cyaneus* ZEA17I, showed species-specific differences on germination and plant growth of cultivated rocket, lamb lettuce, lettuce and tomato. Although we did not detect significant differences on seed germination in vitro, inhibition of lamb lettuce and tomato germination was detected in vivo after the treatment with *S. albidoflavus* VT111I and *S. cyaneus* ZEA17I. This might have been due to the production of some secondary metabolites, as these two strains also reduced the growth of lamb lettuce radicles. Another possibility is that the complex interactions among the antagonist, the natural microflora and the host plant might have transiently negatively affected seed germination. Indeed, negative effects have been sometimes observed after application of BCAs on the germination and plant growth in the absence of the pathogen [55]. However, this negative effect might be mitigated during the growth of the host plant, as we did not see any reduction in the biomass dry weight after bacterization by the three antagonists after 2 weeks of growth..

## Conclusion

The standardized dual culture assay proposed in this work, in which the distance between the streptomycete and pathogen was set as a function of the mycelial growth of fungal pathogens, enabled us to compare the inhibition activity of candidate antagonistic bacteria against multiple pathogens showing different mycelial growth. The selected strains - *S. anulatus* CMJ58I, *S. albidoflavus* VT111I and *S. cyaneus* ZEA17I - showed species-specific effects on plant germination and growth. They only rarely negatively influenced plant growth, and these effects did not persist during the further development. Therefore, they are valuable candidates for the development as BCAs. However, further in vivo studies are needed to evaluate their potential as biological control or plant growth promoting agents.

## Methods

### *Streptomyces* strains

The strains were part of a collection of endophytic streptomycetes maintained in the laboratory of Plant Pathology at the Department of Food, Environmental and Nutritional Sciences (DeFENS), University of Milan, Italy [3]. Strains were grown on Czapek-Yeast Extract medium (CZY: 35 g/L Czapek Dox broth, Difco Laboratories, USA; 2 g/L yeast extract, Difco Laboratories, USA; 15 g/L agar, Applichem, Germany) for 3 weeks at 24 °C. Spores were collected in 10 % sterile glycerol + 0.01 % tween20 and filtered through two layers of sterile gauze. The concentration was determined and the spore suspension was stored at −20 °C.

### Pathogen strains

The fungi and oomycetes used in this work are representatives of the main soil borne pathogens of horticultural crops. *Sclerotinia sclerotiorum* FW361*, Fusarium oxysporum* f.sp. *lactucae* L74*, Thielaviopsis basicola* FW406 and *Pythium ultimum* FW407 belong to a collection maintained in the laboratory of Plant Pathology at DeFENS, University of Milan, Italy. Two additional species, *Rhizoctonia solani* FW408 and *Phytophthora* sp. FW409, were kindly provided by Dr. Andrea Minuto (Centro di Sperimentazione e Assistenza Agricola, Albenga, Italy). The pathogens were maintained at 20 °C on Malt-Extract Agar medium (MEA: 30 g/L malt extract, Difco Laboratories, USA; 15 g/L agar, Applichem, Germany), whereas *Phytophthora* sp. FW409 on V8 medium (200 mL/L V8 Vegetable juice, Campbell food, Belgium; 2 g/L CaCO_3_, Carlo Erba, Italy; 15 g/L agar, Applichem, Germany).

### Pathogen mycelium growth curves

The pathogen agar-mycelium disc (6 mm diameter), taken from the edge of an actively growing fungal colony, was inoculated upside down in the center of the Petri plate containing CZY or Potato Dextrose Agar medium (PDA: 39 g/L Potato Dextrose Agar, Difco Laboratories, USA) and plates were incubated at 24 °C in the dark. Four perpendicularly radial mycelium measurements were taken from the edge of the disc daily for 7 days in three replicates and the mean daily mycelial growth was calculated.

### Dual culture assay

The antibiosis assay was performed in Petri plates (90 mm diameter) containing CZY medium for all fungi and oomycetes, except *T. basicola* FW406, for which PDA was used. The pathogen was inoculated as described above in the center of the Petri plate, whereas 10 μL of streptomycete agar-spore suspension (10 μL of 10^8^ CFU/mL in 90 μL 0.01 % agar) were uniformly distributed along a 40 mm line. The two microorganisms were inoculated the same day at 20 mm and 10 mm distance for group 2- and 3-pathogens, respectively. For the group 1-pathogens, the streptomycetes were inoculated first, and the fungus was inoculated in the center of the plate 2 days after the streptomycete inoculation at 25 mm distance. Three replicates were prepared for each strain and plates inoculated only with the pathogen were used as control. Following the inoculation, plates were incubated at 24 °C in dark. The antagonistic activity was expressed as the percentage of mycelium growth inhibition compared to the control. It was calculated by the formula: (R1-R2)/R1 × 100, where R1 and R2 were the mycelial radial growth of the pathogen in the control and in the presence of the antagonist, respectively.

In the preliminary study, the dual culture assays were performed in order to optimize the timing of inoculation of both microorganisms. *Streptomyces rochei* CMJ57I and *S. anulatus* CX14W were used as reference antagonist strains and *S. sclerotiorum* FW361 and *F. oxysporum* f.sp. *lactucae* L74 as representative pathogens. The antagonists were inoculated on CZY 3, 2 or 1 day before or at the same day as the pathogen and the percentage of inhibition was calculated at 3 and 7 days after pathogen inoculation, respectively.

### Selection of streptomycetes based on their antagonistic activity

One hundred and thirty streptomycete strains were randomly chosen from the collection and their antagonistic activity against the six fungal soil borne pathogens was calculated as described above. The inhibition was assessed 3, 6 or 7 days after pathogen inoculation for group 1, 2 and 3, respectively. Subsequently, rank position was attributed to each streptomycete based on its inhibition activity against each pathogen (from 1 for the most active, to 130 for the least active) and for each strain the average of the six rank positions was calculated. The mean rank values were sorted from the smallest to the highest and the first three strains were chosen for the subsequent experiments (Table 2, Additional file 1: Table S1).

### Scanning electron microscope (SEM) studies of *Streptomyces* seed colonization

The ability of three selected streptomycete strains (*Streptomyces anulatus* CMJ58I, *S. albidoflavus* VT111I and *S. cyaneus* ZEA17I) to colonize seed teguments was assessed under SEM (Leo Electron Microscopy, Cambridge, UK). Seeds of cultivated rocket (*Eruca sativa* Mill.; Fratelli Ingegnoli, Italy), lamb lettuce (*Valerianella locusta* L. ‘Accent’; Enza Zaden, Italy), lettuce (*Lactuca sativa* L. ‘Bionda ricciolina’; Fratelli Ingegnoli, Italy) and tomato (*Solanum lycopersicum* L. ‘Marmande’; Fratelli Ingegnoli, Italy) were surface sterilized in 0.7 % sodium hypochlorite for 5 min and rinsed three times in sterile water. Subsequently, they were immersed in 1 mL of streptomycete spore suspension (10^8^ CFU/mL) and air-dried under the laminar flow hood. Control seeds were treated with sterile water (untreated control). The seeds were then placed on moist filter paper in a Petri dish (20 cm in diameter) and were incubated at 20 °C in dark. Samples were freeze-dried 24, 48 and 72 h after seed inoculation as described previously [56, 57] and observed by SEM.

### Effects of streptomycetes on seed germination and seedling growth in vitro

Three strains, *Streptomyces anulatus* CMJ58I, *S. albidoflavus* VT111I and *S. cyaneus* ZEA17I, were selected based on the results of the dual culture assay. Seeds of cultivated rocket (*Eruca sativa* Mill.), lamb lettuce (*Valerianella locusta* L. ‘Accent’), lettuce (*Lactuca sativa* L. ‘Bionda ricciolina’) and tomato (*Solanum lycopersicum* L. ‘Marmande’) were surface sterilized as described above, immersed in a sufficient amount of *Streptomyces* spore suspension (10^8^ CFU/mL) and air-dried under the laminar flow hood. Control seeds were treated with sterile water (untreated control). One hundred seeds were placed on moist filter paper in three replicates and incubated in the growth chamber at 24 °C, 55 % relative humidity and 15 h photoperiod. During the experiment, filter paper was maintained humid adding a suitable volume of sterile water. Germination was assessed at different times based on vegetable species (Table 3) and the mean germination percentage was calculated. At the end of the experiment, which varied according to vegetable species, root and hypocotyl lengths were measured and the means were calculated.

### Effect of streptomycetes on plant biomass dry weight

Seeds of cultivated rocket (*Eruca sativa* Mill.), lamb lettuce (*Valerianella locusta* L. ‘Accent’), lettuce (*Lactuca sativa* L. ‘Bionda ricciolina’) and tomato (*Solanum lycopersicum* L. ‘Marmande’) were surface sterilized and bacterized with *Streptomyces anulatus* CMJ58I, *S. albidoflavus* VT111I or *S. cyaneus* ZEA17I as described above. Control seeds were treated with sterile water (untreated control). Two experiments were performed. In the first one, 100 seeds in three replicates for every combination plant species - *Streptomyces* were sown in the non-sterilized Irish and Baltic peat based growing substrate (Vigorplant, Italy) in plastic boxes (10 × 10 × 10 cm) and watered with tap water. The plants were grown in the growth chamber at 24 °C, 55 % relative humidity and 15 h photoperiod and watered as necessary with tap water to keep the soil moist. The number of germinated plants was assessed after 10 days of growth for cultivated rocket, lettuce and tomato, and after 14 days for lamb lettuce. After 2 weeks (in case of cultivated rocket, lettuce and tomato) or 3 weeks in case of lamb lettuce, the plants were carefully removed from the boxes and the growing substrate was carefully removed from roots. Fifty plantlets were counted (except lamb lettuce, where 30 plantlets were counted), left to air-dry at room temperature and the biomass dry weight was determined.

In the second experiment, the bacterized seeds of four vegetable crops were placed individually in polystyrene seed trays (48 cm3/cell) watered with tap water. The plants were grown in the growth chamber as described previously. After 3 weeks (except lamb lettuce, which was grown for 4 weeks), the plants were carefully removed from the seed trays and the growing substrate was carefully removed from roots. The plants were left to air-dry at room temperature and the biomass dry weight was determined.

### Production of compounds involved in plant growth promotion activity

#### Indole-3-acetic acid (IAA)

Ten μL of *Streptomyces* spore suspension were inoculated in 5 mL of Czapek broth added with 500 μg/mL of L-tryptophan (Sigma-Aldrich, USA). After 10 days of incubation at 26 °C with constant shaking at 125 rpm in the dark, the production of IAA was determined as described previously [58].

#### Siderophores

Ten μL of *Streptomyces* agar-spore suspension were inoculated in the centre of a Petri plate (90 mm diameter), containing the Fe-free Czapek agar medium prepared as previously described [3]. After 14 days of incubation at 24 °C in the dark, the *Streptomyces* colony was overlaid by 15 mL of the chrome azurol S agar [59] as proposed by Pérez-Miranda et al. [60]. After 1 day of incubation at room temperature in the dark, the change of color around the colony (from blue to orange) indicated the siderophore production.

### Statistical analyses

The statistical analyses were performed using R software, version R3.0.2 [61]. The percent data of inhibition activity and seed germination were arcsine root-square transformed and were submitted to ANOVA. Similarly, ANOVA was performed for data of root and hypocotyl lengths, and biomass dry weight, followed by a Tukey *post-hoc* test for multiple comparison (*P* = 0.05) using the TukeyC package [62].

## Additional file

Additional file 1:
**Table S1.** Mycelial growth inhibition of six soil borne pathogens by 130 *Streptomyces* strains. (DOCX 36 kb)
