# Supplementary material for: Selection of Streptomyces against soil borne fungal pathogens by a standardized dual culture assay and evaluation of their effects on seed germination and plant growth
Source: BMC Microbiol. 2016 Nov 9;16:272. doi: 10.1186/s12866-016-0886-1 (PMC5103511; doi:10.1186/s12866-016-0886-1)
Supplement: Additional file 1: — Table S1. Mycelial growth inhibition of six soil borne pathogens by 130 Streptomyces strains. (DOCX 36 kb) [file 12866_2016_886_MOESM1_ESM.docx]

**Additional file 1: Table S1:** Mycelial growth inhibition of six soil borne pathogens by 130 *Streptomyces* strains

| *Streptomyces* sp. | Medium Rank position value | Mycelium growth inhibition (%) | | | | | |
| --- | --- | --- | --- | --- | --- | --- | --- |
|  |  | *Sclerotinia  sclerotiorum*  FW361 | *Rhizoctonia solani*  FW408 | *Fusarium oxysporum* f.sp. *lattucae* L74 | *Thielaviopsis basicola* FW406 | *Pythium ultimum* FW407 | *Phytophtora* sp. FW409 |
| ZEA17I | 12.0 | 79.49 | 67.54 | 34.06 | 51.79 | 50.74 | 62.00 |
| CMJ58I | 21.8 | 46.79 | 41.75 | 28.99 | 76.19 | 57.35 | 74.42 |
| VT111I | 27.3 | 64.10 | 53.95 | 28.47 | 52.38 | 55.88 | 26.00 |
| SW35W | 32.0 | 52.88 | 39.82 | 33.12 | 45.33 | 48.08 | 56.36 |
| RBF10R | 33.0 | 39.85 | 48.18 | 24.09 | 54.44 | 38.46 | 66.10 |
| SW01W | 33.2 | 54.81 | 38.05 | 31.17 | 60.00 | 29.49 | 60.00 |
| EP07W | 36.0 | 54.81 | 41.59 | 15.58 | 41.33 | 51.52 | 61.82 |
| SW37W | 36.8 | 52.84 | 57.14 | 18.60 | 43.18 | 56.06 | 37.04 |
| MR02W | 38.0 | 44.95 | 47.81 | 0.00 | 83.33 | 44.12 | 65.12 |
| SLF27R | 38.8 | 46.90 | 72.41 | 47.86 | 28.89 | 33.94 | 55.56 |
| ARN02R | 39.2 | 35.92 | 24.53 | 32.50 | 63.10 | 39.39 | 65.25 |
| SG04W | 39.5 | 46.59 | 45.13 | 24.03 | 78.67 | 10.26 | 60.00 |
| MR11W | 40.3 | 53.98 | 47.62 | 0.00 | 80.77 | 49.24 | 51.85 |
| MR01W | 40.7 | 46.15 | 18.27 | 27.54 | 85.71 | 27.94 | 67.44 |
| QR29W | 40.8 | 64.10 | 31.48 | 28.00 | 38.10 | 45.00 | 52.00 |
| ARF24R | 41.3 | 68.35 | 50.93 | 27.01 | 0.00 | 47.50 | 50.00 |
| ALC03R | 42.0 | 65.37 | 56.48 | 29.93 | 33.33 | 41.25 | 12.00 |
| OCB21R | 42.0 | 74.75 | 40.91 | 29.93 | 16.67 | 30.77 | 61.82 |
| VRN01R | 43.2 | 45.71 | 43.52 | 28.00 | 73.81 | 13.75 | 52.00 |
| FS02W | 43.8 | 61.36 | 60.53 | 0.78 | 23.30 | 53.85 | 61.82 |
| AST32I | 44.0 | 62.86 | 56.14 | 9.42 | 45.24 | 49.26 | 9.30 |
| QR26W | 44.0 | 46.15 | 42.54 | 0.73 | 67.86 | 55.88 | 55.81 |
| CRM05R | 44.0 | 44.32 | 42.86 | 13.18 | 43.59 | 46.21 | 56.60 |
| MR24W | 44.0 | 50.96 | 41.59 | 7.75 | 68.00 | 21.79 | 61.82 |
| RMX14R | 45.2 | 30.68 | 69.52 | 42.07 | 61.54 | 6.82 | 52.83 |
| CN08W | 45.3 | 60.55 | 42.59 | 36.00 | 20.00 | 42.50 | 29.09 |
| ER18A | 46.3 | 54.29 | 46.49 | 14.67 | 42.86 | 25.00 | 50.00 |
| PO07W | 46.7 | 51.43 | 43.82 | 18.67 | 34.52 | 46.25 | 50.00 |
| SJS02R | 46.8 | 34.09 | 39.05 | 15.50 | 76.92 | 37.12 | 54.55 |
| RFB14A | 47.0 | 38.64 | 56.58 | 13.18 | 60.00 | 39.74 | 22.22 |
| VLA11R | 48.7 | 47.71 | 32.58 | 24.00 | 57.14 | 32.50 | 44.00 |
| QR16W | 49.0 | 43.81 | 33.71 | 9.86 | 61.90 | 22.50 | 64.00 |
| FA01W | 49.7 | 35.80 | 42.59 | 13.18 | 53.85 | 34.85 | 55.56 |
| ALP11R | 49.7 | 53.98 | 51.42 | 5.71 | 35.71 | 38.18 | 55.56 |
| CU07W | 49.8 | 33.52 | 39.05 | 30.23 | 46.15 | 13.64 | 64.15 |
| GC03W | 50.0 | 64.10 | 42.59 | 6.67 | 45.24 | 23.75 | 50.00 |
| MR19W | 50.2 | 53.85 | 44.25 | 0.00 | 64.00 | 16.67 | 60.00 |
| LMP64I | 50.3 | 51.28 | 42.72 | 26.09 | 35.71 | 48.72 | 13.95 |
| EW15W | 50.5 | 55.68 | 44.29 | 34.48 | 23.08 | 0.00 | 66.04 |
| SYS13R | 51.3 | 47.19 | 18.42 | 29.66 | 38.46 | 34.09 | 56.60 |
| LM01W | 51.3 | 41.35 | 46.02 | 6.49 | 65.33 | 15.38 | 58.18 |
| SLP02R | 52.3 | 71.29 | 27.27 | 25.55 | 41.11 | 14.81 | 50.00 |
| SG12W | 52.5 | 58.25 | 30.19 | 28.75 | 60.71 | 7.88 | 37.29 |
| ZEA07I | 53.8 | 53.85 | 44.74 | 32.85 | 36.90 | 29.41 | 0.00 |
| OCB07R | 53.8 | 44.04 | 43.06 | 24.00 | 35.71 | 12.00 | 63.64 |
| RSM08R | 54.3 | 4.85 | 49.06 | 38.75 | 64.44 | 0.00 | 55.51 |
| OCB15R | 54.5 | 36.70 | 35.96 | 29.58 | 56.82 | 52.56 | 0.00 |
| TOR65I | 55.3 | 58.42 | 41.57 | 0.00 | 74.36 | 47.50 | 0.00 |
| TOR01L | 55.3 | 73.27 | 22.52 | 0.00 | 54.76 | 50.00 | 20.00 |
| EQS04R | 56.0 | 42.31 | 17.70 | 20.93 | 38.67 | 38.46 | 58.18 |
| VT041R | 58.2 | 59.05 | 35.96 | 38.67 | 9.52 | 15.00 | 38.89 |
| VT101I | 58.3 | 56.44 | 51.92 | 0.00 | 41.67 | 20.59 | 34.00 |
| FT04W | 59.8 | 55.45 | 28.07 | 6.57 | 38.10 | 33.75 | 50.00 |
| VT334R | 60.0 | 0.00 | 43.52 | 5.80 | 61.90 | 39.38 | 48.00 |
| PRT07R | 60.0 | 28.41 | 38.10 | 19.77 | 36.54 | 34.62 | 52.73 |
| QR06W | 61.2 | 35.23 | 43.36 | 22.48 | 36.00 | 9.62 | 58.18 |
| FIC35R | 61.3 | 67.33 | 46.36 | 35.77 | 33.33 | 5.13 | 0.00 |
| EPH21R | 62.0 | 67.05 | 44.44 | 17.50 | 43.33 | 9.09 | 0.00 |
| PTH08R | 63.0 | 52.97 | 12.73 | 8.03 | 74.36 | 23.08 | 22.22 |
| CHL01R | 63.8 | 56.64 | 16.67 | 4.65 | 31.82 | 38.46 | 56.36 |
| GC02W | 64.2 | 17.31 | 26.99 | 20.16 | 41.33 | 28.21 | 58.18 |
| ALP07R | 66.0 | 86.89 | 15.09 | 7.14 | 46.43 | 20.00 | 13.56 |
| BT28W | 66.2 | 53.85 | 20.61 | 6.57 | 42.86 | 21.25 | 46.00 |
| RSM10R | 66.2 | 19.32 | 42.98 | 8.57 | 38.64 | 25.64 | 52.73 |
| CR13A | 66.2 | 58.42 | 26.36 | 6.57 | 40.00 | 37.18 | 10.17 |
| CX08W | 66.3 | 26.70 | 50.00 | 0.00 | 36.36 | 63.64 | 22.22 |
| BT29W | 66.3 | 42.72 | 39.62 | 11.25 | 53.33 | 9.70 | 25.93 |
| CPB11R | 67.0 | 62.83 | 34.72 | 0.00 | 42.22 | 37.18 | 0.00 |
| TRX03R | 67.2 | 32.18 | 40.91 | 8.03 | 38.46 | 19.23 | 52.54 |
| LM08W | 67.5 | 29.55 | 42.86 | 2.33 | 53.85 | 6.82 | 58.49 |
| SJ01W | 68.2 | 28.41 | 37.14 | 17.44 | 44.87 | 1.28 | 58.18 |
| ST06W | 69.3 | 40.87 | 20.35 | 6.49 | 44.00 | 12.73 | 61.82 |
| CN09W | 69.7 | 40.00 | 9.71 | 30.43 | 35.71 | 33.82 | 16.28 |
| LMP72I | 70.0 | 17.95 | 35.96 | 31.39 | 37.50 | 35.29 | 0.00 |
| CPB08R | 70.0 | 65.14 | 29.21 | 8.00 | 28.57 | 13.75 | 29.63 |
| PRV04R | 70.2 | 62.14 | 16.98 | 2.50 | 53.57 | 10.30 | 30.51 |
| CN06W | 70.8 | 29.00 | 7.02 | 25.71 | 35.90 | 27.27 | 52.83 |
| AM10A | 71.5 | 63.72 | 12.96 | 22.50 | 48.72 | 6.41 | 5.08 |
| EP05W | 72.5 | 48.57 | 25.44 | 10.22 | 28.57 | 38.13 | 12.00 |
| HYP03R | 73.2 | 0.97 | 9.43 | 10.00 | 38.89 | 30.30 | 61.11 |
| CM20A | 73.5 | 40.00 | 32.58 | 4.23 | 40.48 | 17.50 | 44.00 |
| SYS01R | 74.2 | 28.41 | 40.00 | 33.79 | 34.62 | 15.15 | 0.00 |
| ALG06R | 74.3 | 37.50 | 47.86 | 0.00 | 56.67 | 18.18 | 1.85 |
| TOR57I | 75.2 | 44.95 | 7.30 | 9.33 | 32.14 | 30.00 | 50.00 |
| CN05W | 75.3 | 28.41 | 20.00 | 21.71 | 34.62 | 27.27 | 30.19 |
| ARN09R | 75.5 | 13.59 | 33.02 | 1.25 | 59.52 | 38.79 | 11.11 |
| CRC04R | 76.0 | 52.22 | 27.03 | 0.00 | 52.38 | 9.09 | 38.89 |
| TAG17R | 76.3 | 58.42 | 32.73 | 6.57 | 12.22 | 5.77 | 53.70 |
| CN13W | 76.7 | 46.32 | 58.77 | 11.63 | 28.00 | 6.41 | 3.64 |
| SN02A | 76.7 | 51.46 | 25.47 | 21.25 | 40.48 | 4.24 | 10.17 |
| LRS40R | 77.5 | 26.14 | 37.14 | 3.10 | 0.00 | 24.24 | 62.96 |
| ST07W | 77.7 | 25.00 | 24.78 | 24.81 | 28.00 | 28.21 | 20.00 |
| HYP23R | 79.2 | 0.00 | 0.00 | 6.25 | 51.11 | 29.70 | 56.78 |
| TOR51L | 79.5 | 39.11 | 10.91 | 24.09 | 25.64 | 7.69 | 55.56 |
| HLP08R | 79.7 | 66.50 | 34.91 | 0.00 | 45.24 | 3.03 | 5.93 |
| ARF07R | 79.8 | 19.80 | 30.91 | 8.03 | 35.90 | 5.77 | 59.32 |
| QR19W | 80.2 | 31.82 | 27.14 | 3.88 | 34.62 | 30.30 | 28.30 |
| EP11W | 81.5 | 44.95 | 13.59 | 11.59 | 21.43 | 33.82 | 12.79 |
| ARN08R | 82.7 | 27.18 | 0.00 | 27.50 | 35.71 | 24.24 | 18.52 |
| RMX44 | 84.0 | 45.30 | 17.54 | 16.79 | 16.67 | 30.88 | 0.00 |
| FIC11R | 84.0 | 23.30 | 3.64 | 18.25 | 46.67 | 11.52 | 18.52 |
| LRS20R | 84.3 | 41.28 | 19.10 | 28.00 | 22.62 | 15.00 | 3.64 |
| PRT06R | 84.3 | 47.71 | 3.60 | 0.00 | 19.23 | 36.25 | 50.00 |
| CX17W | 84.5 | 22.73 | 25.93 | 31.25 | 26.67 | 10.00 | 12.00 |
| SG09W | 85.3 | 0.00 | 3.70 | 9.33 | 71.43 | 0.00 | 50.00 |
| VNC12R | 85.5 | 18.61 | 4.63 | 17.33 | 15.38 | 41.25 | 23.64 |
| ROS77F | 86.0 | 47.62 | 2.78 | 9.33 | 30.95 | 25.00 | 10.00 |
| CRM31R | 86.0 | 23.86 | 0.00 | 33.79 | 62.22 | 0.00 | 0.00 |
| CPB02R | 86.8 | 31.07 | 0.94 | 7.50 | 60.71 | 9.09 | 16.95 |
| SW42W | 87.5 | 43.27 | 21.24 | 7.79 | 22.67 | 13.46 | 20.00 |
| PLR02R | 88.8 | 55.94 | 18.18 | 0.73 | 25.64 | 9.62 | 16.95 |
| EPH36R | 90.2 | 21.78 | 7.27 | 18.25 | 46.67 | 6.41 | 8.47 |
| KAL01R | 90.5 | 65.35 | 25.45 | 0.00 | 23.08 | 14.10 | 0.00 |
| TXB01R | 90.8 | 40.59 | 29.09 | 24.09 | 23.08 | 4.94 | 0.00 |
| TOR16L | 91.2 | 13.86 | 6.36 | 34.375 | 0.00 | 11.11 | 20.34 |
| EP03W | 93.0 | 50.44 | 0.00 | 12.50 | 0.00 | 10.00 | 14.81 |
| LRS45R | 93.5 | 6.49 | 15.73 | 13.33 | 29.76 | 16.25 | 12.00 |
| BT08A | 93.5 | 38.46 | 33.63 | 0.00 | 32.00 | 10.26 | 12.73 |
| ALP10R | 93.5 | 27.18 | 11.32 | 2.86 | 60.71 | 4.24 | 11.02 |
| LMN06R | 93.7 | 39.45 | 4.63 | 13.33 | 19.23 | 2.50 | 45.45 |
| ER19A | 94.5 | 49.52 | 5.26 | 5.33 | 33.33 | 9.877 | 8.00 |
| MR16W | 96.3 | 23.86 | 30.95 | 0.00 | 40.00 | 0.00 | 16.98 |
| QR03W | 98.5 | 39.42 | 30.97 | 6.49 | 0.00 | 0.00 | 16.36 |
| SUA02R | 98.5 | 37.62 | 28.18 | 2.19 | 0.00 | 8.97 | 16.95 |
| RBF05R | 99.5 | 41.35 | 8.85 | 6.49 | 24.00 | 4.49 | 12.73 |
| HLC02R | 99.7 | 20.45 | 3.64 | 2.19 | 33.33 | 12.82 | 27.12 |
| SW46W | 99.8 | 20.19 | 7.08 | 6.49 | 25.33 | 17.31 | 12.73 |
| VTV06R | 101.5 | 47.52 | 14.81 | 3.65 | 23.08 | 3.85 | 3.39 |
| CSM12R | 102.5 | 9.71 | 5.66 | 2.86 | 38.10 | 13.94 | 5.56 |
| LRS17R | 106.2 | 13.46 | 21.24 | 7.79 | 14.67 | 7.05 | 4.55 |
